# Dr. Powell's Case of Hydrophobia

**Published:** 1808-09

**Authors:** 


### Dr. POWELL's CASE OF HYDROPHOBIA.

ANN CHANDLER, æt. 31, the mother of three children, the youngest two months old and at the breast, a woman of slight make and delicate habit, was employed in her apartment about eleven in the forenoon of Saturday, July 2, 1808, when a strange cat entered it, and immediately ately fell upon a cat belonging to her. She assisted and released her own cat, and the strange one flew at her and fastened upon her right wrist, where it remained fixed for several minutes, until her screams had called up a man from the yard, who suffocated it by squeezing the neck, before it could be compelled to let go its hold.

The cat was known to have belonged to a person in the neighbourhood, and I therefore endeavoured to trace the source of its affection. Near three weeks before, a dog of the same house had been destroyed on a suspicion of madness: he was pulling the cat about that morning, but was not known to have bit her; no suspicion was therefore entertained: but early in the morning of the day on which she bit Mrs. Chandler, the servants had driven her out of the house, on account of a peculiarity and wildness which alarmed them. About three weeks before, the dog, above alluded to, had been attacked and bit severely in the street, by another which was supposed to be mad, and was killed accordingly.

The report of the existence of rabid animals had from this circumstance been previously, but very vaguely, prevalent in the neighbourhood; and, in consequence of it, she made application to some professional gentlemen, who judged, from the depth the teeth had penetrated, and the situation of the wound, near the radial artery, that no excision could be made, with effect, short of amputation of the arm: and, the circumstances related to them respecting the animal, were so indistinct, as not to justify much alarm, or so formidable a proposition. They therefore washed the wound, and directed her to keep it wet with aqua lithargyri acetati composita; and another surgeon afterwards recommended it to be poulticed. She went also to Margate, and bathed four times in the ordinary way, as an out-patient of the Infirmary there.

The wound had bled very little at first, and healed readily, leaving two small eschars on each side of the carpal extremity of the radius. She continued, however, to feel a sort of heat and trifling uneasiness in the part, which frequently recalled it to her recollection, though she never felt alarmed by it, or apprehensive as to the event.

On Thursday, July 28, the bitten part became hotter, and looked red and rather inflamed, and the pain spread more up the arm. It continued to increase in extent rather than violence, without any redness or swelling of the arm, as far as her own observation went, and at her supper on Sunday, 31st, she afterwards remembered that she did not take her porter without some difficulty. In all other respects pects she was much as usual, and passed the former part of that night without inconvenience or apprehension.

About three o'clock in the morning of August 1, (the 31st day from the bite,) she felt thirsty and uncomfortable, and got up for the purpose of taking some water. On reaching the vessel which contained it, she was seized with a sudden inexplicable repugnance, and compelled to put it from her; about eight she did take a small portion of tea with great effort, and, after that, her aversion to liquids became more and more insurmountable; but she suffered no noticeable inconvenience in any other respect. She had only swallowed a little jelly and a few currants through the day, and had been fully aware of her situation from the first occurrence of the hydrophobia. She had still continued to suckle her child.

At twelve at night I was desired to see her by Mr. Luke Hodgson, when she gave me the preceding history of circumstances with much precision, and firmness and calmness of mind. The bitten part looked rather red just round the eschars, but not to any extent beyond it, and less so in Mr. Hodgson's opinion than it had done in the earlier part of the day; it was not tender when pressed upon, and no part of the arm was swoln or inflamed. A pain extended from the wound up the outer side of the arm to the shoulder and right side of the neck, and there occupied the extent of that half of the trapezius muscle, not affecting in the least the left side of the spine. The glands in the axilla were not enlarged or tender; the skin was cool and natural; the pulse 110, and hurried; the respiration natural, except that there was occasional involuntary sighing, which did not appear to arise from any mental despondence; the tongue was clean; the bowels and urinary organs acted regularly; and she expressed no pain any where, neither in the throat nor in the epigastric region when it was pressed upon.

I offered her a glass of water without previous notice, and she instantly started in alarm and distress to her husband on the opposite side of the bed, but it was only alarm, she had no convulsion, nor did she refer it to any particular local sensation; I afterwards splashed some on the floor out of her sight, and she manifested much inconvenience at the sound of it; she swallowed with visible effort small bits of currant jelly, and said confidently that she should be able to take pills. She had had no sleep since the attack in the morning, neither had she felt any disposition to it. Because all the modes of practice which recurred to my recollection had been unsuccessful after the appearance ance of hydrophobia; because I had a conviction founded on experience, that the nitrate of silver is a most powerful medicine in some other diseases of irritability and irregular convulsion; and because it could be conveniently and safely given in the form of pill, I determined upon trying its effect in the present instance, and accordingly directed that she should take at first one grain every hour.

At nine in the morning of August 2, she was described to have passed a sleepless night, with frequent and violent fits of anxiety and loud screaming from slight causes, but most especially from flies settling on her face; this had been more strongly the case between five and six in the morning, when she could scarcely be held in her bed, and she had often prayed Mr. Hodgson to release her from her horrors by bleeding her to death. Her friends had not been able to meddle with any liquid in the room on account of the effect it produced, and which now appeared considerably increased; she had passed two natural motions and urine freely, without feeling that agitation from its sound or proximity which other liquids excited; this peculiarity, it may here be observed, continued to a more advanced period of the case; for when the noise of tea-cups, or the mention of any sort of fluid which was connected with drinking, excited the greatest effect, she passed her urine, and heard the sound as it passed, without the same inconvenience. About this time also another source of irritation was noticed, for currents of air, as the door of her apartment opened, then first became distressing to her, and this afterwards increased considerably. She had taken her pills regularly, and then took one in a small portion of jelly with hurry and effort, and expressing a desire to yield to the repugnance she felt, and to delay the attempt till it should have passed by.

Anticipating the more violent symptoms which might be expected, I urged her removal to St. Bartholomew's Hospital, near which she lived, and she and her friends yielded to the proposition without hesitation; this was effected with difficulty, though she was carried in mens' arms, on account of the wetness of the streets, and the idea of water excited by the trampling of feet in the mud.

When she had got to bed she appeared calm and comfortable; she was placed in a room separated from the ward, and great attention paid to remove every source of irritation. The pain in the neck had become more troublesome, and she could scarcely bear it to be touched. I took the opportunity of wishing her to point out its boundary, to give her a looking glass, and she did it leisurely, without any any inconvenience from the reflection from its surface. She expressed her desire to be supported constantly upright in bed, for that through the morning she had uniformly felt much choaking in the throat, and anxiety, if she lay down and shut her eyes. Her pulse at that period (half past eleven) was reduced to 90, her skin natural, breathing free, but still with the occasional interruption of sighing, her tongue clean, and protruded without difficulty, and her mind calm, except when under the agency of external irritations. The dose of nitrate of silver was increased to a grain and half every hour, and directions were given for its subsequent further increase.

I had directed that a clyster of strong broth, with one hundred drops of tinctura opii, should be injected; but to this, as bringing a liquid near her, she most anxiously objected, and it was not therefore urged.

Until three o'clock the paroxysms had only recurred about once an hour, and lasted at each attack nearly a minute; they much resembled those of regular hysteria, and as they increased afterwards in their duration, assumed more closely its characters. She described their commencement to be in the stomach, with a working and fulness there, and that a pricking substance passed up into her throat and choked her; she screamed suddenly, and grasped firmly hold of her attendants, as if voluntarily, and muscular convulsions came on, which were sometimes more, sometimes less general and violent.

The causes from which these paroxysms arose were extremely slight: the passage of a fly near her face, the attempt to swallow a pill, a stream of air, the sight of oil or wine, or any other liquids, even the sound of water, and other such circumstances, were sufficient; she now also complained of inconvenience from the light, which was accordingly moderated, and she expressed an anxious desire not to be left.

The tension and fullness of her breasts, and the discharge of milk from them, had become troublesome to her; they were therefore drawn by a syringe, and she expressed some distress at the first moment of its application, from the idea (as she explained herself) of liquid connected with it; but when the exhauster was once fixed this almost ceased.

The effect of sounds was peculiar, for though in the subsequent stages their influence became more general, at this period the effect was rather proportionate to the ideas they excited in her mind than to their violence; bells and other strong noises did not agitate her, but the clatter of earthen earthen ware, the noise of a distant pump, or any thing connected with liquids, produced the paroxysms in all their violence.

She ate a very little sweatbread boiled and then dried, and swallowed some fresh currants, the latter with less resistance than any other substance, providing carefully to pick them dry from the stalk, for if, from any breach of the skin, they happened to be wet, she rejected them with quickness and distress.

Hitherto her mind had been perfectly calm and composed during the intervals, and her conversation and behaviour most correct and proper; her conviction of the fatal termination of the case, and the sufferings she underwent, were supported with the temperate resignation and religious dependance of a superior mind; she wished to spare her connections the distress of witnessing that misery which they could not relieve, and at the same time expressed her affections towards them warmly.

From four o'clock, the impossibility of her swallowing, even a pill, was so marked, and a convulsive paroxysm so certainly the consequence of the offer, that no more were attempted to be given; and she had then taken in the whole fifteen grains, without any sensible effect.

Between four and five she had three fits, which were much stronger than the former, and one of them continued with violent muscular exertion for a quarter of an hour. She described the rising from the stomach to the throat, and a heaving and fullness as from distension by wind as preceding them, and her irritability to external objects had increased, both in its degree and objects: the light, the noise of a door shutting, the wind, even the breath of those who held her, became sources of distress. The pain shot from the back of the neck round to the angles of the jaws, the chin, and throat, the muscles of which however were not affected, except in the general distortion of the paroxysms; for when she was complaining of the violence of this pain, she could open her mouth and put out her tongue. She kept the arms of some of the attendants in a continued and firm grasp, from which she expressed satisfaction, and scarcely loosed them during the intervals.

Between five and six the paroxysms became more frequent, and their dependence upon external causes was less evident; indeed they might then be said to take place almost spontaneously, and they were attended by more restless impatience and loud screaming, and were more violent in all respects; seven such were noted within the hour.

The

The sensations in her stomach and throat entirely left her, in the region of which there had at no time been any inconvenience produced by pressure. She looked pale and exhausted, and, after the paroxysms, there was a tremor and a blueness about the lips and fingers, and the pulse was weaker and more rapid; she began to complain of great soreness and tenderness about the scalp of the head, and the touch of it was immediately productive of convulsions. With these latter attacks she had considerable eructations of wind, from which she expressed herself relieved for the moment; and at half past five she first spat up a small quantity of thick viscid saliva, which she made strong voluntary efforts to keep clear of those around her, often anxiously expressing that she would not hurt any one if she could help it. Her urine passed involuntarily in the bed, and in considerable quantities, and distressed her exceedingly; her skin had become wet with perspiration from the exertions she underwent, but at this she expressed no alarm; her mind and senses were still active and quick, she heard and noticed observations made at a distance in a low tone of voice, and urged impatiently the departure of some of her friends who were near, but she was more and more irritable and uncontrolable.

From six to eight she passed in almost constant convulsions, varying in their degree of violence, with extreme irritability, and impatient of every thing about her; the accidental mention of wine threw her into agony; she complained of failure in her sight; and urged frequently and earnestly her desire to be bled to death; she expressed herself quickly and with effort, using fewer words, and those interruptedly; she had not only threatened violence towards the attendants, and that she would bite them, but had struck them vehemently more than once. She had copious eructations of air, and the secretion of viscid saliva had increased considerably, and was excreted, but with much convulsive effort; she was asked as to the affection of the stomach and throat, which she said had entirely left her; the general perspiration continued, and the urine passed copiously and involuntarily during the convulsions; the pulse was less distinct, weaker, and variable from 140 to 150.

About eight she was herself conscious that she was unmanageable, and had expressed the necessity for more restraint; she had struck and torn the clothes of those who were immediately about her, and had bit Mr. Wheeler in the hand, because he resisted her repeated wish of bleeding her to death, though to him she had previously, throughout, throughout, expressed her gratitude. Fortunately, the cuticle was scarcely grazed, and that part of it was immediately removed by excision. She also bit the nurse in the covered part of the arm, and left a strong impression of her teeth, but they had not perforated the clothes. The confinement of a waistcoat became on these accounts necessary, and was had recourse to.

From this period she had lost all control over her mind, and continued for almost four hours in a paroxysm of furious insanity. About half past eight she called violently for water, and when it was brought, overcame by an effort, the convulsive repugnance she visibly felt at the moment, and took it with eagerness, spilling a good deal at the same time over her, without seeming to notice it; she did this twice, and swallowed in the whole near half a pint, which in a few seconds was returned by vomiting, mixed with mucus and a greenish fluid. She also became desirous for air; and when the window was opened, expressed no inconvenience from the current. Her raving became incessant, with much invective and coarseness of language, and her efforts to release herself from restraint were violent and constant. The waistcoat, in a short time after its application, became a leading source of distress, and she used both rage and persuasion, with all the character of insanity, to get liberated from it, occasionally bartering and promising obedience, and to endeavour to drink, if she was released. She retained a perfect knowledge of those around her, and alive to external circumstances of every kind. The eructations continued to a considerable degree, sometimes accompanied by hiccough, and twice she also vomited some thin greenish fluid. For a long time her exertions appeared to consist in regulated endeavours to release herself, and not to be involuntary convulsions; but about eleven o'clock they again resumed their former character. At that time she took two spoons full of porter, which was rejected immediately; some tinctura opii was also given in cinnamon water, and rejected. The abdomen had become tense and swoln; the convulsions continued, but weaker; and she remained in this state without any violent exertion. The circumstances of her disease seemed, however, to be strongly impressed upon her mind, for she recapitulated the whole of them in a rambling and incoherent way.

At about half past twelve she sunk into an unconscious state, with slighter general muscular twitchings only, and a rapid indistinct pulse, laborious respiration, cold sweats, and coldness of extremities. From this she seemed to rally rally at a quarter before two, and complained of light from the candle; she took some cinnamon water with tincture of opium, but immediately vomited it, with an increase of convulsion; and died at two o'clock, forty-seven hours after the first marked occurrence of Hydrophobia.

### Examination of the Body.

The vessels of the brain and its membranes were much loaded with blood, and nearly half an ounce of colourless fluid was contained in the ventricles.

The intestines were much distended by air, and the peritonæum and also the pleura were in a state of unusual and remarkable dryness; there was more fluid than natural in the pericardium.

The œsophagus was contracted, and strongly marked by longitudinal rugæ; it was somewhat redder than natural, and over its whole internal surface there was spread a thin transparent membrane-like unorganized layer of coagulable lymph.

The trachea was in a natural state.

The stomach contained nearly half a pint of greenish fluid; and upon its internal surface, which was rather redder and more vascular than natural, there were, under the internal membrane, some small spots of extravasated blood collected into clusters; these were not numerous, and were chiefly found about the cardia.

The body had been previously removed to her own dwelling, and therefore we had not opportunity of making a very minute examination.

*Essex Street, Strand, August* 15, 1808.

